# Performance of an Indirect Packed Bed Reactor for Chemical Energy Storage

**DOI:** 10.3390/ma14185149

**Published:** 2021-09-08

**Authors:** Tiziano Delise, Salvatore Sau, Anna Chiara Tizzoni, Annarita Spadoni, Natale Corsaro, Raffaele Liberatore, Tania Morabito, Emiliana Mansi

**Affiliations:** 1 ENEA-Italian National Agency for New Technologies, Energy and Sustainable Economic Development, SSPT-PROMAS-TEMAF Technical Unit for Renewable Energy Sources, Via Ravegnana 186, 48018 Faenza, Italy; tiziano.delise@enea.it; 2 ENEA-Italian National Agency for New Technologies, Energy and Sustainable Economic Development, TERIN-STSN-SCIS Technical Unit for Renewable Energy Sources, Casaccia Center Rome-Via Anguillarese 301, SM di Galeria, 00123 Roma, Italy; annachiara.tizzoni@enea.it (A.C.T.); annarita.spadoni@enea.it (A.S.); natale.corsaro@enea.it (N.C.); raffaele.liberatore@enea.it (R.L.); 3 Department of Chemical Engineering for the Sustainable Development, University Campus Bio-Medico di Roma, Via Álvaro del Portillo, 21, 00128 Roma, Italy; tania.morabito@hotmail.it; 4 ENEA-Italian National Agency for New Technologies, Energy and Sustainable Economic Development, FSN-FISS-SNI Laboratory of Innovative Nuclear System, Casaccia Center Rome-Via Anguillarese 301, SM di Galeria, 00123 Roma, Italy; emiliana.mansi@gmail.com

**Keywords:** thermal storage, thermochemical energy storage, indirect heat exchanger, packed bed, fluid-dynamic simulations, storage efficiency

## Abstract

Chemical systems for thermal energy storage are promising routes to overcome the issue of solar irradiation discontinuity, helping to improve the cost-effectiveness and dispatchability of this technology. The present work is concerned with the simulation of a configuration based on an indirect-packed bed heat exchanger, for which few experimental and modelling data are available about practical applications. Since air shows advantages both as a reactant and heat transfer fluid, the modelling was performed considering a redox oxide based system, and, for this purpose, it was considered a pelletized aluminum/manganese spinel. A symmetrical configuration was selected and the calculation was carried out considering a heat duty of 125 MWth and a storage period of 8 h. Firstly, the heat exchanger was sized considering the mass and energy balances for the discharging step, and, subsequently, air inlet temperature and mass flow were determined for the charging step. The system performances were then modelled as a function of the heat exchanger length and the charging and discharging time, by solving the relative 1D Navier-Stokes equations. Despite limitations in the global heat exchange efficiency, resulting in an oversize of the storage system, the results showed a good storage efficiency of about 0.7.

## 1. Introduction

The design of feasible thermal storage systems (TES) is a key point to allow complete commercialization and diffusion of the CSP (Concentrating Solar Power) technology, making it possible to overcome the irregularity of solar energy availability. In this regard, thermochemical accumulation (CS TES), where the enthalpy of a single reversible reaction is used for heat charge and discharge, is very promising among the several methods proposed and developed in the scientific literature [1,2,3,4,5].

Numerous chemical compounds have been investigated in this context, including oxides/hydroxides couples [6,7,8,9,10,11], carbonates/oxides [12], and processes involving reduction/oxidation cycles. The latter presented a very interesting prospect allowing to use air both as reactant and heat transfer fluid (HTF) [13]. Several oxides and mixed oxides are proposed at this aim [14,15,16,17],with the general purpose to select systems with good energy density and stability under the charging/discharging cycles. However, little is reported in the scientific literature about the applications of these materials in real conditions, as an alternative to the currently employed sensible heat storage systems.

Actually, to provide practical applicability for these types of TES, they need to be supported and/or synthesized with an appropriate geometry and size. Moreover, it is also necessary to design a suitable heat exchanger configuration, to ensure an efficient energy transfer between the thermochemical storage system and the heat transfer fluid during the charging and discharging processes.

Currently, most works are related to the oxide/hydroxide systems, with direct and indirect heat exchange geometries including fluidized and packed beds [8,9,10,11,18,19,20,21,22,23,24]. As far as oxide redox couples are concerned, structured fixed-bed systems are reported, based on cobalt oxides, perovskites and doped mixed oxides, and with direct contact between the TES and the HTF [16,25,26,27,28,29]. Although this configuration is advantageous concerning energy transfer efficiency, it presents limitations about the allowable HTF mass flow, which is related to the storage thermal power. In fact, the pressure drop inside a packed bed is plainly a function of the particle size and the HTF linear velocity [30]; therefore, a decrease in the former parameter leads to an increase in energy density but also to constraints for the maximum permitted HTF flow rate. As a consequence, unless unrealistic geometries are considered with very large flow sections and short reaction/heat transfer paths, it can be supposed that an indirect configuration could be preferable for packed beds. With this concept, the TES and reacting gas are separated from the HTF, and the mass flow and velocity of the latter can be optimized with respect to thermal exchange efficiency. For this purpose, Esaki et al., at low-temperature levels [31], and Shaube et al. for the CaO/Ca(OH)_2_ couple [10], described a configuration based on a plates geometry, where pressurized air is used as charging/discharging HTF, while the TES reactions occur on the other side of the heat exchanger.

So far, no similar configurations were proposed for applications at temperatures above 500 °C; therefore, the purpose of this work was to analyze the fluid-dynamic behavior of a heat exchanger/reactor employing an oxide based thermochemical system. In this respect, it was necessary to consider a TES synthesized and characterized as pellets of proper size, and, for this purpose, a non-toxic and cost-effective manganese aluminum spinel was selected [30].

The design of a suitable configuration has to account for a modular arrangement which allows an easy scaling for the heat exchanger. Following this aim, a symmetrical geometry was selected, consisting of evenly spaced linear tubes passing through the TES fixed bed. The fluid-dynamic simulation was carried out assuming 125 MWth of duty, pressurized air as HTF and considering the discharging phase, strictly related to the final user requirements, as the step that determines the reactor/heat exchanger size. The HTF temperature and mass flow for the charging step were then calculated accordingly.

## 2. Materials and Methods

To obtain significant data from the simulation activities, it was necessary to consider stable pelletized thermochemical TES materials, with available experimentally validated thermophysical data. For this reason, a manganese/aluminum spinel was selected, according to the work of Morabito et al. [30], which presents low cost and toxicity along with a discharging temperature that allows to operate with very efficient heat/electricity power conversion blocks. The TES charging/discharging cycles are temperature-controlled, meaning that both processes are carried out at the same oxygen pressure [30], following the reaction:

(1) $$Mn{Al}_{2}O_{4}⇌Mn{Al}_{2}O_{4-\delta }+\frac{\delta }{2}O_{2}.$$

The TES thermophysical and kinetics properties are reported in Table 1 [30].

Air was assumed both as reactant through the packed bed at about 1 bar (HTF2), and, according to the scientific literature for similar applications [32], pressurized at 10 bar as heat transfer fluid in the heat exchanger channels (HTF1). Regarding the powder side, the charging process occurs only above 700 °C, while discharging happens from 500 to 650 °C, but with good reaction rates only above 600 °C [30]. Everything considered, the powder temperature operating range was set from 600 to 790 °C, including a relevant part of sensible heat.

As the discharging phase is bound to the requests for the power block, its sizing determines the overall dimensions of the heat exchanger. In this work, a highly efficient Rankine cycle, operating within a 530 °C and 240 °C temperature interval [33,34], was considered as the final user. As a consequence, HTF1 inlet and outlet temperatures were bounded to, respectively, 290 and 550 °C. Reactive air (HTF2) was set to enter the fixed bed at 1.1 bar and 600 °C, with a mass flow rate calculated at 1.5 times the stoichiometric oxygen demand in reaction 1, in order to maintain the oxygen partial pressure at the level necessary for the oxidation process. Table 2 summarizes the input and the averaged thermophysical properties values for HTF1 and HTF2. As for the latter, they are calculated considering a 600–790 °C interval.

**Figure 1:**
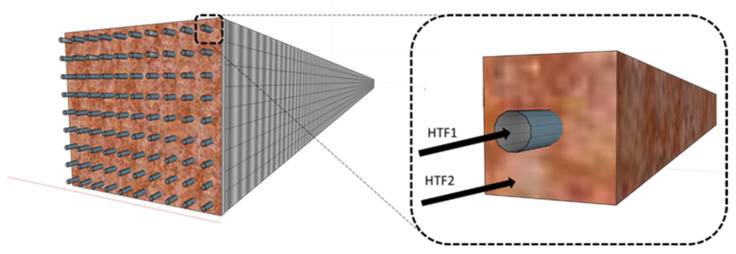
Tridimensional geometry of the heat exchanger/reactor. The whole system is produced by the translation of a repetitive section, shown on the right. HTF1 represents the air flowing inside the central tube, while HTF2 indicates the reactant air passing through the TES.

Concerning the geometry of an indirect heat exchanger coupled with the TES reactor (), the main criterion was to consider configurations realistic for eventual manufacturing processes, and similar to what was described in the scientific literature. For this purpose, it was assumed a symmetrically spaced tube bundle passing through a tank containing the TES was assumed. The system is schematized in Figure 1 and it is similar to a pilot-scale plant reported in the scientific literature for oxide/hydroxide couples, where the heat exchanger conduits are organized alternatively to work as TES oxidation or reduction reactor [10].

**Figure 2:**
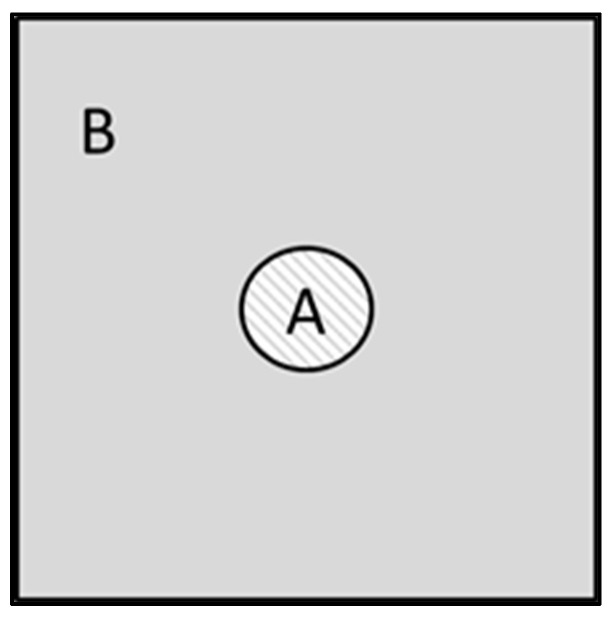
Repetitive section on the xy plane of the geometry described Figure 1: in the (**A**) channels flow the heat transfer fluid (pressurized air) and (**B**) is the tank zone containing the TES pellets (MnAl_2_O_4_).

It is possible to simplify the fluid-dynamic simulation by considering that the whole heat exchanger can be described by translating in the xy plane (being z the direction of the HTF flow) the repetitive section illustrated in Figure 2, where A indicates the channels used to flow the heat transfer fluid (HTF1) and B the zone containing the TES. A homogeneous temperature was assumed in each powder section at constant z, and the phenomena along the external borders were not considered. To calculate the heat exchange coefficient on the TES part, an average thickness was defined (and used in Equation (8a)), according to the following equation, with L as the side length of the repetitive section and r_A_ the radius of the HTF1 conduit:

(2) $$\mathrm{Bside}=\frac{\left(\frac{L}{2}-r_{A}\right)+\left(\sqrt{2{\left(\frac{L}{2}\right)}^{2}}-r_{A}\right)}{2}.$$

The determination of the equivalent diameter for the HTF1 channel (employed in Equation (11a)) was straightforward:

(3) $$D_{h\_\mathrm{HTF}1}=2r_{A}.$$

The actual free surface on the HTF2 side (used in Equations (7) and (11b)) was calculated by the expression below:

(4) $${\mathrm{Surf}}_{\mathrm{HTF}2}=\varepsilon \left(L^{2}-{\pi r}_{A}{}^{2}\right).$$

The mass and energy balances necessary for the sizing of the heat exchanger are described in Table 3. The equations system was solved using the fminimax MATLAB (R2020a) function, adopting the constraint to operate with a flow above the laminar regime, that is, with a Reynolds number not smaller than 5000 on the HTF1 side [36]. The HTF2 heat exchange behavior during the discharging phase was described considering a direct contact packed bed, as reported by Xu et al. [37,38]. A 125 MWth user was considered, with an accumulation period of 8 h, typical of nightly storage for real size CSP plants [33,34].

The following assumptions were considered:

○ The powder behaves as a thermocline and transversal heat fluxes (along the reactor length) are considered negligible [18]. This is a realistic assumption based on literature data on thermal storage with solids for CSP [39,40,41];
○ The whole system is considered as adiabatic;
○ The heat resistance of the intermediate wall of the reactor is negligible due to its small thickness;
○ TES and HTF enthalpy and temperature change only along the z-axe direction of the HTF1 flow;
○ The oxygen concentration on the powder TES side is considered constant throughout the discharging phase.

This way it was possible to settle a minimum volume for the heat exchanger, taking into account the geometry parameters and powder amount necessary for the energy balances and the user requirement. Then, to determine a more realistic sizing for the TES system, it was necessary to consider the actual TES/HTF1 heat transfer behavior over the charging and discharging periods and the HX length, carrying out a fluid-dynamic simulation, and using as starting values the results of the previous calculation. At this aim, each material (TES, HTF1 and HTF2) was associated with the correspondent scalar 1-D energy Navier–Stokes equation [42,43] as reported in Table 4.

Sreact is the reaction rate per volume unit, which can be obtained by a chemical controlled Shrinking Core model [30], and assuming the powder side of the heat exchanger as a plug flow reactor. The derivation of the expression for the reaction rate per volume and time is illustrated in Appendix A and resulted:

(19) $$R_{i}\;\left[\frac{\mathrm{mol}}{{\mathrm{sm}}^{3}}\right]=\;r\frac{3}{R_{\mathrm{part}}}\frac{\rho_{\mathrm{app}}}{\rho_{\mathrm{bulk}}},$$

leading to

(20) $$\mathrm{Sreact}=R_{i}\cdot H_{r}.$$

The Matlab pdepe function was used to solve the Navier–Stokes equations within a time and length (t, z) 1-D mesh grid, considering Equations (13)–(15) as elliptical partial differential expressions. Finally, the discharging storage efficiency can be calculated by the following equation:

(21) $$\eta =\frac{\mathrm{minimum}\;\mathrm{heat}\;\mathrm{exchanger}\;\mathrm{size}\;\left(\mathrm{volume}\right)}{\mathrm{actual}\;\mathrm{heat}\;\mathrm{exchanger}\;\mathrm{size}\;\left(\mathrm{volume}\right)}.$$

## 3. Results

Although possible long-term applications cannot be ruled out for thermochemical materials, these systems can be more advantageously assumed for short term storage applications, utilizing both the powder reaction enthalpy and sensible heat. In fact, a thermochemical TES must be maintained at the reaction temperature until the beginning of the discharging step. This result can be realistically achieved only for storage periods not longer than a few days after the charging process, unless an external energy backup is inconveniently employed. For instance, the material considered as TES in this work presents an onset discharging point of around 600 °C [30]; since the powder thermal capacity is equal to 800 J/(kg·K) [30], 460 kJ/kg of energy would be required to bring the spinel from room temperature up to that value, which is greater than the 133 kJ/kg of discharging enthalpy [30].

**Figure 3:**
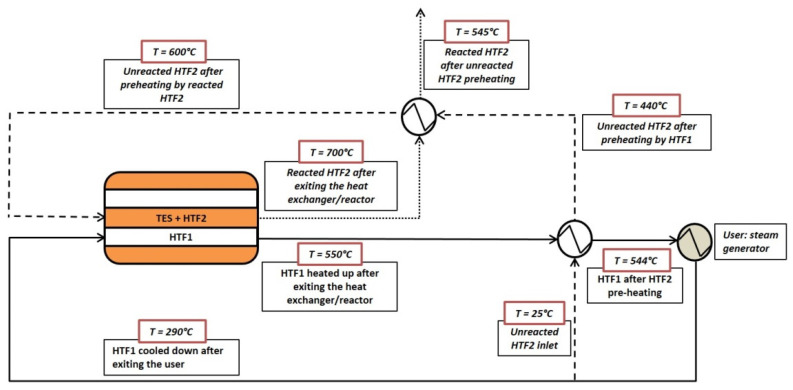
Scheme of the discharging process. The continuous line represents the HTF1 stream, the dashed line the HTF2 before reacting and the pointed line the HTF2 after the oxidation (discharging) process.

As described above, a first calculation step was carried out to determine the geometry parameters corresponding to a minimum heat exchanger volume, ensuring the mass and energy balances. The discharging process is schematized in Figure 3. After the charging step, the TES is initially at 790 °C and its final temperature is set at 600 °C. Then, compressed air (HTF1) flows from the outlet of the user (a steam generator for a Rankine cycle) inside the exchanger. At the same time, a more limited air mass flow (HTF2) is introduced at 600 °C (to avoid the cooling of the pellets) through the TES to achieve the oxidation reaction in the range between 600 and 650 °C. The sensible heat absorbed by the reactant gas from the powder is quite small and requires to increase the TES mass of around 3%. Since most of the HTF2 oxygen is employed for the reaction, it cannot be recycled, and thermal power must be continuously provided to heat the reactant air up to 600 °C. This can be attained by two heat exchangers placed upstream the powder inlet. In the first one, the HTF1 stream exiting the heat exchanger is utilized; since the HTF2 mass flow is relatively very low (about one hundredth) in comparison with the value presented by HTF1, the latter cools down only a few degrees, heating HTF2 from room temperature to 440 °C. The remaining sensible heat, up to 600 °C, might be provided by the reacted HTF2, which would cool from 770 to about 630 °C.

The actual procedure to solve the equations in Table 3 was as following:

○ A minimum radius of about one-third of an inch (0.072 cm) was set for the heat transfer tube (r_A_), to avoid significant pressure drops with the expected HTF1 linear velocities, according to the data obtained with online calculators [44];
○ Given the assumption that the powder cross-section is isothermal, the B-side (L) parameter cannot be too much large and, for this reason, a maximum value of about 2 inches was conservatively considered in the calculation;
○ The three geometry parameters: r_A_, L (and, consequently, B_side_ from Equation (2) and the HX length were varied and for each value, and then:
  ▪ A Reynold number of 5500 was conservatively imposed;
  ▪ HTF1 velocity was therefore also determined from Equation (11a);
  ▪ The correspondent HTF1 mass flow and the overall heat exchange coefficient were then calculated;
○ It was checked if, with these imposed and calculated values, Equations (5)–(7), (14a) and (15) were all verified together. If not, the geometry parameters were varied until the required solution is achieved.

As expectable, the convergence of the equations system was obtained for the minimum HTF1 channel diameter. In this case, a maximum value is reached for both the heat exchange coefficient and surface. On the other hand, a decrease in powder thickness improves the overall energy transfer but also reduces the TES amount, and so the energy stored in each transversal section unity. Thus, the smallest total volume was achieved with the maximum value for the powder side (L in Equation (2)).

**Figure 4:**
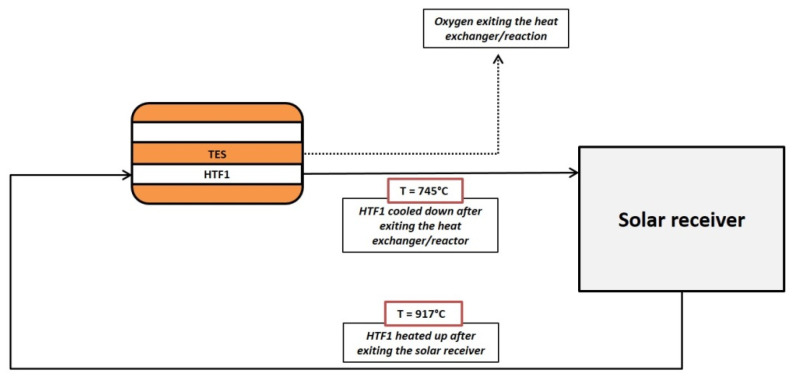
Scheme of the charging process. The continuous line represents the HTF1 stream, the pointed line the oxygen produced by the TES reduction and removed by a pumping system or with a sweeping gas.

It was then possible to define the charging conditions, that is, the HTF1 flow rate and inlet temperature required to bring back the TES at 790 °C and provide the enthalpy to reduce the spinel. The related equations in Table 3 are employed, following the scheme reported in Figure 4. The Reynolds number was bound to be not less than 5000, in order to be behind the transition zone between laminar and turbulent regimes. During this process, hot air is injected into the HTF1 channels, to provide both the necessary sensible and reduction enthalpy, with the latter reactions occurring between 700 and 790 °C. The oxygen produced on the powder side can be removed by a sweeping gas or a pumping system.

Table 5 summarizes the overall outcomes for the two processes, including the resulting size of the repetitive section of Figure 2.

A more realistic heat exchange behavior during discharging was obtained with a fluid-dynamic model, carried out by solving Equations (16)–(18) and using the results of the previous calculation as starting input parameters.

The initial and boundary conditions were set as shown in Table 6. From the experimental data, it is clear that the powder oxidation rate is practically negligible above 650 °C [30], and therefore, only sensible heat can be transferred above that temperature level. The target was to determine the conditions with which the outlet HTF1 temperature resulted of 550 °C over the entire discharging time. For this purpose, an iterative calculation was performed varying HTF1 velocity, the geometry parameters and the heat exchanger length (z_max) until the required output was achieved. Clearly, the results had to comply with the requested heat duty and the balances expressed in Table 3.

**Figure 5:**
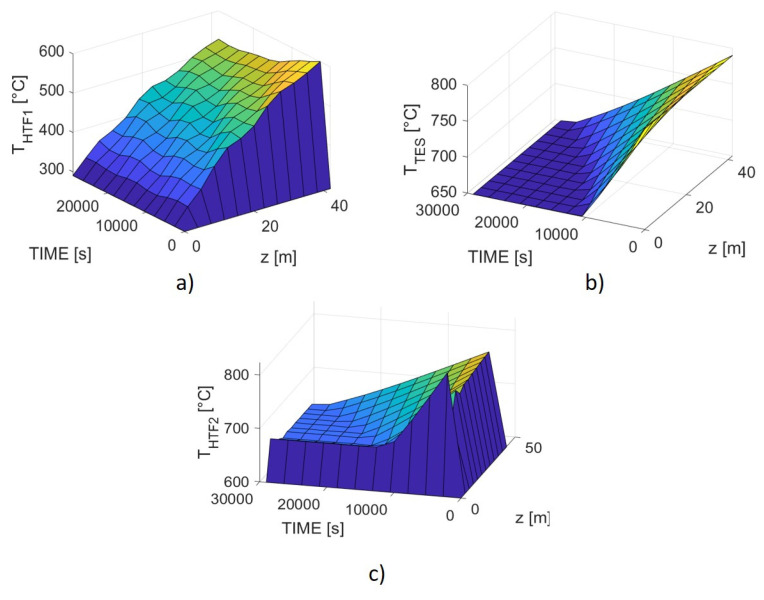
Simulated patterns for the discharging process as a function of HX length and discharging time. (**a**) HTF1 temperature; (**b**) TES temperature; (**c**) HTF2 temperature.

The resulting patterns are illustrated in Figure 5, where the TES, HTF1, and HTF2 temperatures are reported as a function of time and z-length. Once the onset temperature for oxidation is reached, the heat exchange between the powder and HTF1 resulted slower than the exothermic thermal energy accumulation inside the TES, causing an increase of the TES temperature and, as a consequence, a stop for the reaction. Hence, the general pattern is a continuous switch-on/switch-off of the oxidation process, which leads to an isothermal level for the powder, maintained until the end of the discharging period. The HTF1 temperature profile at the maximum length is not regular along the discharging time, anyway, it presents small oscillations around a value of around 540 °C, which is not the targeted level, but still acceptable for the user requirements. Actually, a level of 550 °C could only be acquired by setting an unsuitable heat exchanger length.

The simulation showed that the sensible and reaction enthalpies related to the pellets can be only partially utilizable. In addition, the poor effectiveness of the heat transfer process leads to a significant increase of the heat exchanger length, from 29 to 42 m, and, consequently, of the TES amount. Actually, it is not possible to reduce the system volume employing the remaining reaction enthalpy and sensible heat from 650 to 600 °C, because it would lead to particularly low HTF1 outlet temperatures unless quite unrealistic heat exchanger lengths are assumed. On the other hand, this result allows for a minor temperature reduction to be restored during the charging step.

Regarding HTF2, the reactant air pattern shows a sudden temperature increase at the heat exchanger inlet, with a spike near t = 0 s, that maybe depends on computing problems near the boundary conditions, and the reason may lie in the limited number of points usable for the calculation. However, it could be also a reasonable behavior, considering that at the beginning the reactant gas is in direct contact with the spinel which is still at its maximum temperature level; afterwards, the TES cools down and HTF2 temperature assumes a more regular trend.

As realistically expectable, the HTF2 outlet temperature decreases over the discharging time, from 770 °C to about 650 °C, following the overall cooling of the TES.

It is then possible to estimate a discharging storage efficiency using Equation (21), obtaining a value of 0.69.

Once established the overall size of the heat exchanger, it was possible to define the conditions for the charging step. In particular, it was necessary to set the proper HTF1 inlet temperature and mass flow, necessary to provide the required reaction enthalpy and sensible heat to bring back the TES at 790 °C and to reduce again the spinel. Since no reactant gas is present within the powder side (except a possible sweeping flow), only Equations (16) and (17) were solved together, using the boundary conditions described in Table 7.

**Figure 6:**
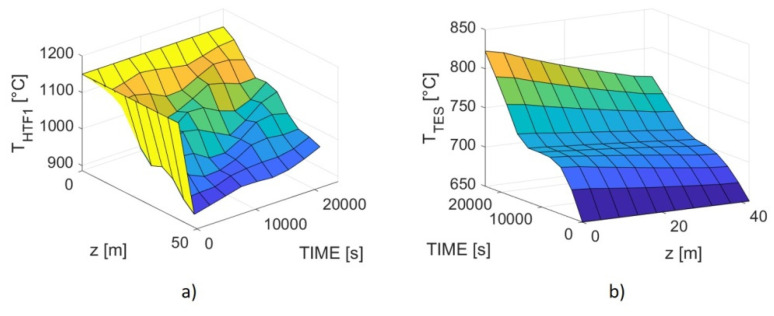
Simulated patterns for the charging process as a function of HX length and charging time. (**a**) HTF1 temperature; (**b**) TES temperature.

Convergence was attained only by setting a high inlet temperature for the pressurized air, namely, 1150 °C, therefore, quite greater than the 917 °C resulting from the Matlab calculation. The outlet HTF1 temperature turned out to be around 930 °C. Actually, it was not possible to solve the equations system imposing lower thermal values, given the necessity to maintain the Reynolds number above 5000, which implies that the pressurized airflow could not be decreased to compensate for the poor thermal exchange behavior. The resulting patterns are shown in Figure 6, showing an overall charging time of 6.7 h; as expected, after having absorbed sensible heat from 650 to 700 °C, the TES remains at the latter temperature until the charging (oxidation) process is completed and, eventually, the powder temperature starts increasing again. It is difficult to make a physical sense of the irregular HTF1 pattern, especially at low time values; at any rate, the overall behavior can be considered realistic, as it highlights the problematics related to a relatively scarce energy transfer between pressurized air and TES.

Table 8 summarizes all the simulation results. Besides a large volume, the storage system requires also large heat exchange surfaces, to be provided by a high number of tubes passing through the pellets. Therefore, all considered, this type of heat accumulation could result more feasible for smaller sizes than the ones considered in this work.

It is noteworthy that kinetics is not a limiting factor, neither in the discharging nor in the charging steps. Actually, both reaction rates can be calculated using Equations (19) and (20), and, taking into account the actual TES mass to be oxidized or reduced, it is possible, using Equations (A14) and (A15) in Appendix A, to demonstrate that the reaction time is always less than 25 s.

## 4. Discussion

To summarize, the fluid-dynamic simulation points out the issues presented by an indirect heat exchange between a solid and a pressurized gas. Actually, despite the very small dimensions of the contiguous channels, and, therefore, the relatively good heat transfer surface, the global storage efficiency resulted significantly different from the unity. In view of practical applications, a geometry suitable for scaling was considered. The same simulation procedure can readily be extended to other thermochemical TES material and more complex configurations, for instance, using finned tubes or different geometries for the channels, where the heat exchange coefficients values can be increased, but, similar issues would expectably arise.

Finally, it is very interesting a comparison between the results of this work and the energy density of other storage systems. The ones based on molten salts sensible heat represent currently the most employed arrangement for CSP plants. In particular, a mixture named “solar salt” (NaNO_3_/KNO_3_ 60/40 wt%) is largely used as TES [45], generally with a two tanks configuration [46]. Commonly, thermal oil is utilized as HTF and the molten salts as TES operating between 390 and 290 °C [47,48]. In other cases, the same “solar salt” is used also as HTF, in a direct and active storage system, exploiting a larger temperature interval, namely from 550 to 290 °C [33]. Evidently, the latter situation presents temperature levels comparable with the system investigated in this work. Using the “solar salt” specific heat and density at the temperatures concerned [34,46,49,50], it is possible to estimate the related volumetric energy density, considering a storage efficiency about unitary for this type of storage system, and neglecting the presence of the intermediate heat exchanger between the two tanks [34].

Concerning other types of energy accumulation, very few data are present in the scientific literature. Xu et al. reported an accurate modelling of a direct contact heat exchanger employing encapsulated PCM based on chlorides [38]. Regarding thermochemical storage, several works have been described, as mentioned in the introduction section, with direct and indirect heat exchangers. However, it is difficult to obtain from the available data the actual sizing of the systems as a function of the heat duty and discharging time. Thus, to roughly evaluate the possible performances of these materials, it can be considered the intrinsic energy density presented by some pelletized compounds. The spinel concerned in this work presents a maximum available energy per volume of about 0.3 GJ/m^3^, while the actual value calculated for the indirect heat exchanger was 0.12 GJ/m^3^. Consequently, it can be calculated another efficiency parameter, defined by the ratio between the actual and intrinsic (maximum) energy densities, attaining a value of 0.42. Then, the size that the indirect heat exchanger would present using other thermochemical systems can be approximately assessed by multiplying this factor with the energy densities of these storage materials. In this regard, Sattler [51] investigated a manganese oxides couple, Funayama et al. [23] the calcium oxide/calcium hydroxide system and Han et al. [24] studied the properties of a structured aluminum/calcium carbonate TES.

Table 9 reports the obtained results, showing similarity among the energy densities obtained with the spinel and the sensible heat system at a temperature range of 100 °C. On the other hand, the HX size is quite lesser if the “solar salt” is used in a larger temperature interval.

Clearly, the configuration with encapsulated PCM corresponds to the most favorable value. Regarding thermochemical storage materials, the performance of the spinel is logically similar to the one presented by the manganese oxide based TES. It would be interesting to consider also other oxides/mixed oxides couples, but no data were found concerning their thermophysical properties in structured or pelletized shapes. Evidently, both hydroxides and carbonates based TES present high reaction enthalpies, and, as a consequence, quite promising volumetric energy densities.

## 5. Conclusions

The purpose of this paper was to model the thermophysical behavior of an indirect thermal energy storage system utilizing a thermochemical storage-based material, also exploitable with respect to its sensible heat. The studied TES was selected mainly considering characteristics of low toxicity and cost along with high availability and reproducible preparation process. Moreover, the storage material must be synthesized in a shape suitable for real-life reactors. For these reasons, the simulation was carried out considering a pelletized manganese aluminum spinel.

The results showed, as expected, that the heat exchange process between the heat transfer fluid and the solid TES represents the most important issue to be optimized for a favorable storage configuration design. In fact, the preliminary sizing of the system, carried out considering mass and energy balances, led to a total length of 29 m, but the fluid-dynamic simulation demonstrated the necessity to oversize the heat exchanger up to 42 m. As a consequence, the discharging storage efficiency could be calculated as equal to 0.69.

Regarding the charging phase, the limited energy transfer properties bounded the pressurized air inlet temperatures to particularly high values, namely, 1150 °C.

The proposed simulation method is relatively straightforward, and it is based on the simultaneous resolution of a one-dimensional equations system. This assumption can be justified by the small size of the assumed heat exchanger sides. Although the model can present some inaccuracy near the inlet of the heat exchanger, the obtained profiles, and the correspondent calculated storage efficiency, can be considered quite realistic and as a good approximation of real situations.

The resulting volumetric energy density (about 0.12 GJ/mol) was found to be comparable to the sensible heat storage systems. Nevertheless, quite higher values could be obtained considering other thermochemical TES materials having greater reaction enthalpies, such as the ones based on calcium carbonate or calcium hydroxide.

To conclude, the advantages of using thermochemical systems compared to molten salts cannot be properly highlighted by mere dimensional comparisons. In fact, solid TES systems can be very easily handled at room temperature, improving the operation and maintenance procedures with respect to molten nitrates, which can require long periods for their fusion. Furthermore, the use of stable solids is also desirable when the storage containers must be filled or emptied. For all these reasons, besides the necessary improvement of their thermochemical features, chemical system materials can be overall proposed as a valid dispatchable alternative for heat storage.

## Figures and Tables

**Table 1:** Thermophysical and kinetic properties of the spinel, from [30].

Powder Channel (TES)			
MW spinel	172.9	$\frac{g}{\mathrm{mol}}$	Molecular weight
R__part_	7.5 · 10^−6^	M	Average radius
δ (from Equation (1))	0.04		Reaction stoichiometric coefficient at Pair ≈ 1 bar
b (calculated as 2/δ)	50		
R__part_	7.5 · 10^−6^	M	Average particle radius
ϱ__app_	4627	$\frac{\mathrm{mol}}{m^{3}}$	Apparent Density
ϱ__bulk_	29,497	$\frac{\mathrm{mol}}{m^{3}}$	Bulk Density
ε	0.83		Fractional void volume
T__TES (min)_	500	°C	Powder initial temperature
T__TES (max)_	790	°C	Powder final temperature
H__r_	23	$\frac{\mathrm{kJ}}{\mathrm{mol}}$	Reaction (oxidation) enthalpy
cp__TES_	138.3	$\frac{J}{\mathrm{mol}\cdot K}$	Specific heat (averaged in the operating temperature range)
k__TES_	0.056	$\frac{W}{m\cdot K}$	Thermal conductivity
Ea (for discharging)	6.5	$\frac{\mathrm{kJ}}{\mathrm{mol}}$	Arrhenius activation energy
Ea (for charging)	425.8	$\frac{\mathrm{kJ}}{\mathrm{mol}}$	
A (for discharging)	5.33 × 10^−6^	$\frac{\mathrm{mol}}{m^{2}\;s}$	Arrhenius pre-exponential factor
A (for charging)	3.83 × 10^16^	$s^{-1}$	

**Table 2:** Discharging phase: HTF1 and HTF2 onput parameters and averaged thermophysical properties [35].

**HTF1**			
T__HFT1_ (in)	290	°C	
T__HFT1_ (out)	550	°C	
k__HTF1_	0.051	$\frac{W}{m\cdot K}$	Thermal conductivity
ϱ__HTF1 (P = 10 bar)_	179.7	$\frac{\mathrm{mol}}{m^{3}}$	Density
C_p,_HTF1_	31.17	$\frac{J}{\mathrm{mol}\cdot K}$	Specific heat gas
µ__HTF1_	0.21 · 10^−3^	Pa·s	Dynamic Viscosity
**HTF2**			
T__HFT2_ (in)	600	°C	
T__HFT2_ (out)	To be calculated	°C	
k__HTF2_	0.064	$\frac{W}{m\cdot K}$	Thermal conductivity
ϱ__HTF2_	66.30	$\frac{\mathrm{mol}}{m^{3}}$	Density
C_p,_HTF2_	32.52	$\frac{J}{\mathrm{mol}\cdot K}$	Specific heat gas
µ__HTF1_	0.26 · 10^−3^	Pa·s	Dynamic Viscosity

**Table 3:** Mass and energy balance equations for discharging and charging steps.

Heat duty	(5) $E=\mathrm{Wuser}\cdot t_{\mathrm{discharging}}$
Necessary powder amount	(6) $$m_{\mathrm{TES}}=\frac{E}{\mathrm{Cp}{\_}_{\mathrm{TES}}\cdot {\Delta T}_{\mathrm{TES}}+H_{r}}+\frac{\left(\frac{F_{\begin{array}{c} \mathrm{HTF}2\_\mathrm{IN} \end{array}}\cdot \mathrm{Cp}{\_}_{\mathrm{HTF}2}\cdot \Delta T_{\mathrm{HTF}2}}{t_{\mathrm{discharging}}}\right)}{\mathrm{Cp}{\_}_{\mathrm{TES}}\cdot {\Delta T}_{\mathrm{TES}}+H_{r}}$$
Necessary heat exchange surface	(7) ${\mathrm{Surf}}_{\mathrm{HTF}2}=\frac{\mathrm{Wuser}}{U_{\mathrm{HTF}1-\mathrm{TES}}{\Delta T}_{\mathrm{HTF}1-\mathrm{TES}}}$
Overall heat exchange coefficients	(8a) $U_{\mathrm{HTF}1-\mathrm{TES}}=\frac{1}{\frac{1}{h_{\mathrm{HTF}1}}+\frac{B_{\mathrm{side}}}{k_{\mathrm{TES}}}}$
	(8b) $$U_{\mathrm{HTF}2-\mathrm{TES}}\approx h_{\mathrm{HTF}2}$$
Logarithmic mean temperature differences	(9a) ${\Delta T}_{\mathrm{HTF}1-\mathrm{TES}}=\frac{\Delta T_{\mathrm{HTF}1}-\Delta T_{\mathrm{TES}}}{\ln\frac{\Delta T_{\mathrm{HTF}1}}{\Delta T_{\mathrm{TES}}}}$
	(9b) $${\Delta T}_{\mathrm{HTF}2-\mathrm{TES}}=\frac{\Delta T_{\mathrm{HTF}2}-\Delta T_{\mathrm{TES}}}{\ln\frac{\Delta T_{\mathrm{HTF}2}}{\Delta T_{\mathrm{TES}}}}$$
HTF1 heat transfer coefficient	(10a) $h_{\mathrm{HTF}1}=\frac{{\mathrm{Nu}\;k}_{\mathrm{HTF}1}}{D_{h\_\mathrm{HTF}1}}$
HTF2 heat transfer coefficient (discharging)	(10b) hHTF2=0.191m˙ReHTF2−0.278cpHTF2PrεπRpart2
Reynolds Number HTF1	(11a) ReHTF1=ρHTF1vHTF1Dh_HTF1µHTF1
Reynolds Number HTF2 (discharging)	(11b) ReHTF2=2RpartFHTF2(1−ε)ρHTF2νHTF2SurfHTF2
Prandtl Number	(12) $\mathrm{Pr}=\frac{{µ}_{\mathrm{HTF}}{\mathrm{Cp}}_{\mathrm{HTF}}}{k_{\mathrm{HTF}}}$
Nusselt Number	(13) Nu=0.023Re0.8Pr13
Energy balance discharging	(14a) $$\begin{array}{c} F_{\begin{array}{c} \mathrm{HTF}1 \end{array}}\cdot {\mathrm{Cp}}_{\_\mathrm{HTF}1}\cdot {\Delta T}_{\mathrm{HTF}1-\mathrm{TES}}+F_{\mathrm{HTF}2\_\mathrm{in}}\cdot \mathrm{Cp}{\_}_{\mathrm{HTF}2}\cdot {\Delta T}_{\mathrm{HTF}2-\mathrm{TES}}\\ =\frac{m_{\mathrm{TES}}\cdot ({\mathrm{Cp}}_{\mathrm{TES}}\cdot {\Delta T}_{\mathrm{TES}}+H_{r})}{t_{\mathrm{discharg}}} \end{array}$$
Energy balance charging	(14b) $$F_{\begin{array}{c} \mathrm{HTF}1 \end{array}}\cdot {\mathrm{Cp}}_{\_\mathrm{HTF}1}\cdot {\Delta T}_{\mathrm{HTF}1-\mathrm{TES}}=\frac{m_{\mathrm{TES}}\cdot ({\mathrm{Cp}}_{\mathrm{TES}}\cdot {\Delta T}_{\begin{array}{c} \mathrm{TES} \end{array}}-H_{r})}{t_{\mathrm{discharg}}}$$
Calculation of the air (in excess) necessary to complete the discharging reaction	(15) $$F_{\begin{array}{c} \mathrm{HTF}2\_\mathrm{IN} \end{array}}=1.5\cdot \frac{\left(\frac{\delta \cdot \frac{m_{\mathrm{TES}}}{{\mathrm{MW}}_{\mathrm{TES}}}}{0.2}\right)}{t_{\mathrm{discharg}}}$$

**Table 4:** Energy Navier–Stokes scalar equations used for the fluid-dynamic simulation.

TES	(16) $\rho_{\mathrm{TES}}C_{p,s,\mathrm{TES}}\frac{\partial }{\partial t}T_{\mathrm{TES}}=k_{\mathrm{TES}}\frac{\partial^{2}}{\partial z^{2}}T_{\mathrm{TES}}+\varepsilon \cdot \mathrm{Sreact}-\left(1-\varepsilon \right)\frac{U_{1}}{s2}\left(T_{\mathrm{TES}}-T_{\mathrm{HTF}1}\right)-\left(1-\varepsilon \right)\;\frac{U_{2}}{s2}\left(T_{\mathrm{TES}}-T_{\mathrm{HTF}2}\right)$
HTF1	(17) $\rho_{\mathrm{HTF}1}C_{p,s;\mathrm{HTF}1}\frac{\partial }{\partial t}T_{\mathrm{HTF}1}=h_{\mathrm{HTF}1}D_{H}\left(\frac{\partial^{2}}{\partial z^{2}}T_{\mathrm{HTF}1}\right)-\rho_{\mathrm{HTF}1}u_{\mathrm{HTF}1}c_{p,g,\mathrm{HTF}1}\;\frac{\partial }{\partial z}T_{\mathrm{HTF}1}+\frac{U_{1}\;}{s1}\;\left(T_{\mathrm{TES}}-T_{\mathrm{HTF}1}\right)$
HTF2	(18) $\rho_{\mathrm{HTF}2}C_{p,s;\mathrm{HTF}2}\frac{\partial }{\partial t}T_{\mathrm{HTF}2}=h_{\mathrm{HTF}2}D_{H}\left(\frac{\partial^{2}}{\partial z^{2}}T_{\mathrm{HTF}2}\right)-\rho_{\mathrm{HTF}2}u_{\mathrm{HTF}2}c_{p,g,\mathrm{HTF}2}\;\frac{\partial }{\partial z}T_{\mathrm{HTF}2}+\varepsilon \frac{U_{2}\;}{s2}\;\left(T_{\mathrm{TES}}-T_{\mathrm{HTF}2}\right)$

**Table 5:** Sizing and operating conditions determined for the storage heat exchanger.

	Discharging	Charging	Units
User storage	125	MWth	
Storage time	7.9	hours	
Total powder mass	12,395	tons	
HTF1 Pressure	10	10	bar
HTF2 Pressure	1.1	1.1	bar
T_TESin_	790	600	°C
T_TESfin_	600	790	°C
T_HTF1in_	290	917	°C
T_HTF1fin_	550	745	°C
T_HTF2in_	500	-	°C
T_HTF2fin_	770	-	°C
A side (*r_A_* from Equation (20))	0.00762		m
L (from Equation (19))	0.05350		m
HX length	29		m
Average powder thickness (Bside from Equation (19))	0.025		m
Global heat exchange surface	334,567		m^2^
Global heat exchanger volume	20,011		m^3^
Overall HTF_1_ mass flow	0.48	0.73	ton/s
HTF_1_ velocity (one channel)	2.35	4.10	m/s
Reynolds numbers for HTF_1_	5500	5100	
Overall average heat exchange coefficient HTF_1_/TES	1.95	2.07	W/m^2^·K
Overall HTF_2_ mass flow	0.011	n.a.	ton/s

**Table 6:** Initial and boundary conditions for the simulation of the discharging step.

INITIAL CONDITIONS	BOUNDARY CONDITIONS	
$T_{\mathrm{HTF}1}$ (z, 0) = 290 °C	T_HTF1_ (0, t) = 290 °C T_HTF1_ (z_max, t) = target: 550 °C	$\frac{\partial }{\partial z}{\;T}_{\mathrm{HTF}1}{\;|}_{z=0}=$ not bounded $\frac{\partial }{\partial z}{\;T}_{\mathrm{HTF}1}{\;|}_{z=z\_\max}=$ 0
$T_{\mathrm{HTF}2}$ (z, 0) = 600 °C	T_HTF2_ (0, t) = 600 °C T_HTF2_ (z_max, t) = not bounded	$\frac{\partial }{\partial z}{\;T}_{\mathrm{HTF}2}{\;|}_{z=0}=$ not bounded $\frac{\partial }{\partial z}{\;T}_{\mathrm{HTF}2}{\;|}_{z=z\_\max}=$ 0
$T_{\mathrm{TES}}$ (z, 0) = 790 °C	$T_{\mathrm{TES}}$ (0,t) = not bounded $T_{\mathrm{TES}}$ (z_max, t) = not bounded $\mathrm{Sreact}=\{\begin{array}{l} R_{i}\cdot H_{r},\;T_{\mathrm{TES}}\leq 650{\;}^{{}^{\circ}}C\\ 0,\;{\;T}_{\mathrm{TES}}>650{\;}^{{}^{\circ}}C \end{array}$	$\frac{\partial }{\partial z}T_{\mathrm{TES}}{\;|}_{z=0}=0$ $\frac{\partial }{\partial z}{\;T}_{\mathrm{TES}}{\;|}_{z=z\_\max}=$ 0

**Table 7:** Initial and boundary conditions for the simulation of the charging step.

INITIAL CONDITIONS	BOUNDARY CONDITIONS	
$T_{\mathrm{HTF}1}$ (z, 0) = to be determined (1150 °C)	T_HTF1_ (0, t) = to be determined (1150 °C) T_HTF1_ (z_max, t) = not bounded	$\frac{\partial }{\partial z}{\;T}_{\mathrm{HTF}1}{\;|}_{z=0}=$ not bounded $\frac{\partial }{\partial z}{\;T}_{\mathrm{HTF}1}{\;|}_{z=z\_\max}=$ 0
$T_{\mathrm{TES}}$ (z, 0) = 650 °C	$T_{\mathrm{TES}}$ (0, t) = not bounded $T_{\mathrm{TES}}$ (z_max, t) = target: 790 °C $\mathrm{Sreact}=\{\begin{array}{l} -R_{i}\cdot H_{r},\;T_{\mathrm{TES}}\geq 700{\;}^{{}^{\circ}}C\\ 0,\;T_{\mathrm{TES}}<700{\;}^{{}^{\circ}}C \end{array}$	$\frac{\partial }{\partial z}T_{\mathrm{TES}}{\;|}_{z=0}=0$ $\frac{\partial }{\partial z}{\;T}_{\mathrm{TES}}{\;|}_{z=z\_\max}=$0

**Table 8:** Heat exchanger/reactor parameters obtained by the fluid-dynamic simulation.

	Discharging Phase	Charging Phase	Unit
B-side	0.048		m
Average powder thickness	0.021		m
r_A_	0.0076		m
Length	42		m
v_HTF1_ (m/s)	2.32	4.44	m/s
Overall heat transfer coefficient HTF1/TES	2.5	2.9	W/m^2^·K
Reynolds Number	5450	5500	
T__HTF1 _IN_	290	1150	°C
T__HTF1 _OUT_	550	900–950	°C
Time	8	6.7	h

**Table 9:** Comparison between the volumetric energy densities obtained with the thermochemical TES studied in this work and the ones presented by other storage systems.

	Volumetric Energy Density (GJ/m^3^)	Pellets Size	Notes
Chemical storage TES system (this work)	0.12	Pellets with average diameter 175 µm	
Sensible heat 390–290 °C (two tanks)	0.13	n.a.	
Sensible heat 550–290 °C (two tanks)	0.34	n.a.	
LiCl-KCl eutectic (390 °C) [38]	1.50	Capsules 4 cm diameter	Direct heat exchanger
Mn_2_O_3_/Mn_3_O_4_ (980 °C) [51]	0.19	Estimated considering open loop/bulk porosity ε = 0.5	Value estimated considering a global efficiency of 0.42
CaO/Ca(OH)_2_ (500 °C) [23]	0.42	Pellets diameter of 1.9 mm and length of 2–10 mm	Value estimated considering a global efficiency of 0.42
CaO/CaCO_3_ (750 °C) [24]	0.89	Pellets with average diameter 150 µm	Value estimated considering a global efficiency of 0.42

## Data Availability

External data are used for the simulation carried out in the article; their sources are duly cited in the text.

## Appendix A

Assuming the powder side as a plug flow reactor:

(A1) $$-F_{O_{2in}}dX=r\;dA_{tot_{particles}}.$$

Considering that *r* is the reaction rate, either for charging or discharging, calculated with the Shrinking core model [26]:

(A2) $$r_{discharging}=-\frac{b}{4\pi R_{c}^{2}}\frac{dN_{TES_{reduced\;state}}}{dt}=bk^{discharging}\;C_{O_{2}},$$

(A3) $$r_{charging}=\frac{b}{4\pi R_{c}^{2}}\frac{dN_{TES_{oxidized\;state}}}{dt}=bk^{charging},$$

where *rc* is the extension of the reaction core (that is, *R_part_*–*R_c_*) and it becomes equal to zero when the reaction is completed. $C_{O_{2}}$ is the oxygen partial pressure of HTF2, that is, on the powder side. Also, $dA_{tot_{particles}}$ is the particles reactant surface contained into a reactor section *dV*, and *X* is the oxygen conversion reaction expressed as

(A4) $$X_{O_{2}}=\frac{F_{O_{2in}}-F_{O_{2out}}}{F_{O_{2in}}}.$$

*t* is therefore necessary to relate $A_{tot_{particles}}$ with the reactor volume. Considering the expression for the apparent and bulk densities:

(A5) $$\rho_{app}=\frac{w_{particles}}{V_{react}},$$

(A6) $$\rho_{bulk}=\frac{w_{particles}}{V_{particles}}.$$

Dividing Equation (A5) by Equation (A6) it is possible to obtain the total volume occupied by the particles:

(A7) $$V_{particles}=\frac{\rho_{app}}{\rho_{bulk}}\cdot V_{react}.$$

On the other hand, the total particles volume is also equal to

(A8) $$V_{particles}=\frac{4}{3}\pi Rpart^{3}\cdot N_{particles}.$$

Inserting Equation (A7) in Equation (A8):

(A9) $$\frac{\rho_{app}}{\rho_{bulk}}\cdot V_{react}=\frac{4}{3}\pi Rpart^{3}\cdot N_{particles}.$$

Morevoer, considering that

(A10) $$A_{tot_{particles}}=4\;\pi Rpart^{2}\cdot N_{particles},$$

it is possible to explicit the *N_particles* value in this way:

(A11) $$N_{particles}=\frac{A_{tot_{particles}}}{4\;\pi Rpart^{2}}.$$

Replacing Equation (A9) in Equation (A7) and differentiating, it follows:

(A12) $$\frac{R}{3}dA_{tot_{particles}}=\frac{\rho_{app}}{\rho_{bulk}}\cdot dV_{react}.$$

Finally, isolating $dA_{tot_{particles}}$ and replacing it in Equation (A2):

(A13) $$-F_{O_{2in}}dX=r\frac{R}{3}\frac{\rho_{app}}{\rho_{bulk}}\cdot dV_{react}.$$

From which it is possible to extrapolate the reaction rate per time and volume that is Equation (A14) (19 in main text):

(A14) $$R_{i}\;\left[\frac{\mathrm{mol}}{\sec\;m^{3}}\right]=r\frac{3}{Rpart}\frac{\rho_{app}}{\rho_{bulk}}.$$

Finally, it is also possible to calculate which is possible to extrapolate the reaction rate per time and volume. In fact, integrating Equations (A3) and (A4) with respect to *rc*, and until the reaction is completed, it is possible to obtain:

(A15) $$\tau_{discharging}=\frac{\rho_{TESmolar\cdot R_{part}}}{r_{discharging}},$$

(A16) $$\tau_{charging}=\frac{\rho_{TESmolar\cdot R_{part}}}{r_{charging}}.$$
